# Highly Stretchable, Self-Adhesive, Antidrying Ionic Conductive Organohydrogels for Strain Sensors

**DOI:** 10.3390/molecules28062817

**Published:** 2023-03-21

**Authors:** Xinmin Huang, Chengwei Wang, Lianhe Yang, Xiang Ao

**Affiliations:** 1 Yancheng Institute of Technology, College of Textile & Clothing, Yancheng 224051, China; 2 School of Textile & Science Engineering, Tiangong University, Tianjin 300387, China

**Keywords:** organohydrogel, double network, antifreezing, antidrying, strain sensor

## Abstract

As flexible wearable devices, hydrogel sensors have attracted extensive attention in the field of soft electronics. However, the application or long-term stability of conventional hydrogels at extreme temperatures remains a challenge due to the presence of water. Antifreezing and antidrying ionic conductive organohydrogels were prepared using cellulose nanocrystals and gelatin as raw materials, and the hydrogels were prepared in a water/glycerol binary solvent by a one-pot method. The prepared hydrogels were characterized by scanning electron microscopy and Fourier transform infrared spectroscopy. The mechanical properties, electrical conductivity, and sensing properties of the hydrogels were studied by means of a universal material testing machine and LCR digital bridge. The results show that the ionic conductive hydrogel exhibits high stretchability (elongation at break, 584.35%) and firmness (up to 0.16 MPa). As the binary solvent easily forms strong hydrogen bonds with water molecules, experiments show that the organohydrogels exhibit excellent freezing and drying (7 days). The organohydrogels maintain conductivity and stable sensitivity at a temperature range (−50 °C–50 °C) and after long-term storage (7 days). Moreover, the organohydrogel-based wearable sensors with a gauge factor of 6.47 (strain, 0−400%) could detect human motions. Therefore, multifunctional organohydrogel wearable sensors with antifreezing and antidrying properties have promising potential for human body monitoring under a broad range of environmental conditions.

## 1. Introduction

In recent years, the application of flexible, wearable, and stretchable electronic devices has received a lot of attention, and they have a wide range of applications in electronic equipment fields, such as human life monitoring signals [1,2], flexible sensors [3,4], electronic skin [5], human robotics [6], and so forth. Hydrogel is a good new wet and soft material with high water content and soft-wet properties in a three-dimensional network [7]. Ionic hydrogel conductors with high-strength stretchability, biocompatibility, and transparency are potential candidates for wearable electronics. However, most flexible conductive hydrogel sensors are completed by the introduction of conductive fillers (such as carbon nanotubes [8], reduced graphene oxide [9], nano-metal particles [10], conductive polymers [11], and so forth). Based on the abovementioned method, the inherent color, and the limited ductility of hydrogel (commonly at 200–400%), the characteristics of the easy aggregation of these conductive components in the polymer network degrade the mechanical properties of this type of conductive hydrogel [12]. In addition, such conductive hydrogels are usually black, and the transparency of the hydrogels is significantly reduced. For a new generation of wearable and flexible electronics, a key performance combination of robust mechanical properties, high sensitivity, visual transparency, and biocompatibility is required. Hydrogels have emerged as a promising material candidate for next-generation bioelectronic interfaces due to their similarities to biological tissues and their versatility in electrical, mechanical, and biofunctional engineering [13,14,15].

Introducing abundant, non-toxic natural polymer materials (such as chitosan (CS) [16], sodium alginate (SA) [17], guar gum [18], and so forth) to prepare flexible conductive materials has become a current research hotspot. Gelatin is a macromolecular hydrophilic colloid and a commonly used natural polymer derived from collagen, which is biocompatible and biodegradable. However, the poor mechanical properties of gelatin-based hydrogels have hindered their development [19]. Chen et al. [20] designed reversible thermal gelatin/polyacrylamide (gelatin/PAAm) double network hydrogels. Hydrogels have good mechanical strength and self-healing properties. However, most hydrogel sensors based on natural polymers lack adhesive properties and require external force to attach to the human body and electronic skin, which leads to unsatisfactory signal detection in human body monitoring. Zhang et al. [21] prepared purely physically crosslinked double network (DN) hydrogel poly(sulfobetaine-co-acrylic acid)/chitosan–citric acid hydrogel. The hydrogel is highly stretchable, transparent, fatigue-resistant, self-adhesive, and self-healing. Cellulose nanocrystals derived from the most abundant native renewable biomass have unique and promising properties, such as sustainability, biocompatibility, a large surface area, and high mechanical strength [22,23,24]. In this regard, cellulose is often combined with inorganic salt ions (such as sodium chloride [25], lithium chloride [26], ferric chloride [9], and so forth) to make conductive nanocomposite complexes, which play an important role in toughening, crosslinking, and acting as a network support with excellent mechanical properties. Song et al. [27] dissolved polyvinyl alcohol (PVA), cellulose nanofibers (CNF), and aluminum chloride hexahydrate (AlCl_3_·6H_2_O) in dimethylsulfoxide (DMSO)/water binary solvents to prepare ionic conductive hydrogels. The resulting ionic conductive organohydrogels exhibited high stretchability (up to 696%), fast response (130 ms), wide operating temperature (−50 °C to 50 °C), and long-term stability (30 days). The introduction of ions can not only provide conductivity to hydrogels but also improve their mechanical properties.

Meanwhile, due to the water molecular structure of the reversible network of the hydrogel sensor, the hydrogel may become ice solid under long-term storage or low-temperature conditions [28,29]. Therefore, this is an important indicator that traditional hydrogel devices need to be used in a relatively mild environment to maintain their original performance, which severely limits the operating temperature range and long-term durability of hydrogel sensors. The existence of the binary solvent system enables the antifreeze hydrogel sensor to lower the freezing point of water in the hydrogel network, suppress the formation of ice crystals, and increase the crosslink density of hydrogen bonds between organic solvents and water molecules [30]. Ni et al. [31] reported a PVA/TA@talc molecular-level ion channel ionic conductive organohydrogel. It had antifreeze and moisturizing properties prepared by a simple EG/water binary solvent dispersion method.

Herein, we designed self-adhesive, highly stretchable, moisturizing, antifreeze, and conductive organohydrogels with strain-sensitive and monitoring properties. First, cellulose nanocrystals (CNC) were used as nanofillers by a simple one-pot method and ultraviolet light irradiation. Double network ionic conductive organohydrogels were prepared by dissolving poly(acrylic acid) (PAA)/gelatin (Gel) double network, metal ions (Al^3+^), and tannic acid (TA) in the dispersion medium of water (H_2_O)/glycerol binary solvent. Glycerol was added to improve the antifreezing performance and water retention performance of the hydrogels, and Al^3+^ and Cl^-^ were incorporated to improve the conductivity of the hydrogels. The introduction of TA gave the organohydrogels good self-adhesive properties. The microstructure of the prepared hydrogel was characterized by scanning electron microscopy (SEM) and Fourier transform infrared spectroscopy (FTIR). The mechanical properties, electrical conductivity, antifreezing properties, water retention properties, and sensing properties of the hydrogels were studied by means of testing methods, such as universal material testing machines and the LCR digital bridge. Strain sensors assembled from organohydrogels exhibit excellent anti-dehydration (7 days), high ionic conductivity (up to 0.14 S/m), and high sensitivity (GF = 6.47). The introduction of free ions (Al^3+^) provides effective conductive ionic channels for organohydrogels, making it possible to detect human activities even at a temperature range (−50 °C to 50 °C) and after long-term storage (7 days).

## 2. Results and Discussion

### 2.1. Preparation and Characterization of the Organohydrogels

**Figure 1:**
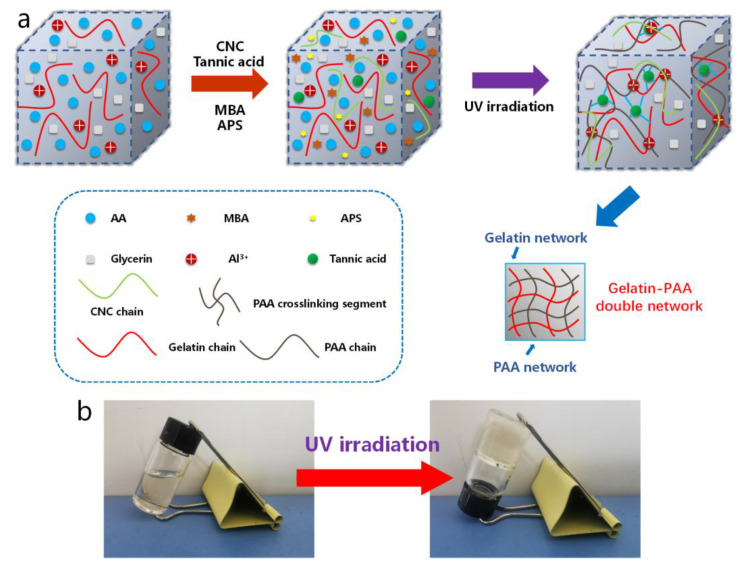
(**a**) Schematic diagram of the process of preparing PAA/CNC/Gel/TA/Al^3+^ double network ionic conductive organohydrogels by one-pot UV illumination. (**b**) Photographs of the organohydrogel preparation process.

As shown in Figure 1, PCGTA organohydrogels were synthesized by a simple one-pot method and photopolymerization (Figure 1a). The interfacial region of organohydrogels is the chain entanglement generated by the hydrogen bond association of gelatin chains [20]. The hydrogels used poly(acrylic acid) (PAA)/gelatin (Gel) double network, tannic acid (TA)/metal ion (Al^3+^) coordination, and water/glycerin binary solvent as the dispersion medium. The transformation to gel was realized after 0.5 h of UV irradiation (Figure 1b). In addition, the introduction of TA as an adhesive gave PCGTA organohydrogels excellent adhesion properties on various surfaces, such as glass, plastic, and metal materials. In this organohydrogel network, the molecular structure of gelatin contains a large number of active functional groups. Intermolecular hydrogen bonding enables gelatin to form a gel (PAA and Gel double network structure) at a certain concentration and temperature; the gelatin molecule is partially crosslinked by tannic acid [32]. Through double crosslinking to enhance their mechanical properties, the interaction between these components enables organohydrogel-based wearable strain sensors to detect, quantify, and monitor motion. The dispersion medium of water/glycerol binary solvent at a temperature range (−50 °C–50 °C) endowed the organohydrogels with excellent antifreeze and antidrying properties, and maintained good strain sensitivity.

### 2.2. Morphology Characterization

**Figure 2:**
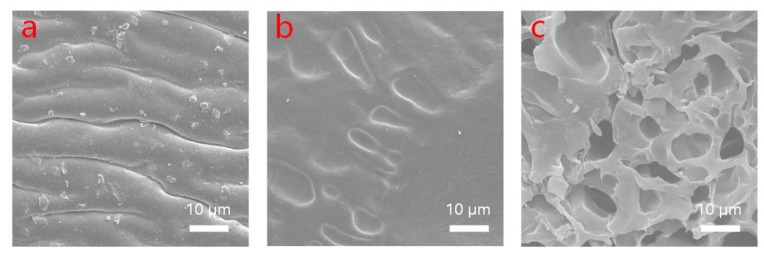
Micromorphological structure of hydrogels (**a**) without glycerol; (**b**) 0% CNC; (**c**) 0.1% CNC.

To explore the microscopic morphology of the hydrogels, the organohydrogels were washed to remove non-lyophilized glycerol. The SEMs of the hydrogels without glycerol or with different CNC contents were characterized. As shown in Figure 2, the glycerol-free hydrogel exhibited a wavy curve (Figure 2a). Moreover, no porous network structure was observed for organohydrogels without CNC (Figure 2b). With the addition of CNC, the organohydrogels exhibited 3D porous network structures. A large number of porous structures regularly exist in this organohydrogel (Figure 2c), while the number of pores is strongly correlated with the TA and CNC crosslink density. The TA and CNC material is connected to organohydrogel through hydrogen bonds [33]. The interconnected porous structures of hydrogel provide a pathway for the movement of conductive ions, which is beneficial to the conductivity.

### 2.3. FTIR Analysis

**Figure 3:**
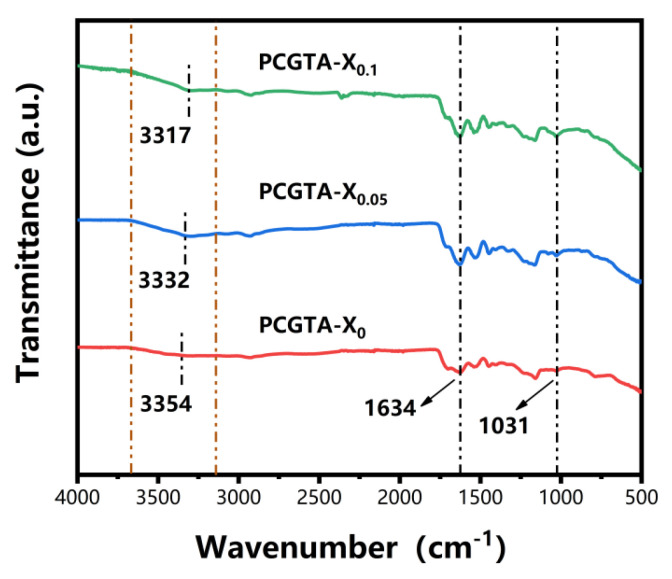
FTIR spectra of PCGTA organohydrogels’ varying concentrations of CNC.

As shown in Figure 3, all spectra contained characteristic absorption peaks in the range of 3150–3650 cm^−1^, which are attributed to the stretching vibrations of -OH bonds. With the increase of cellulose nanocrystals content, the peak of absorption bands of the -OH stretching vibrations exhibited a red shift from a high wavenumber of 3354 cm^−1^ to a low wavenumber of 3317 cm^−1^. This indicates that strong hydrogen bonds were formed between tannic acid, which is rich in phenolic hydroxyl groups, and cellulose nanocrystals [34]. Moreover, the characteristic absorption peak 1634 cm^−1^ is attributed to stretching vibration of C=O bonds [35]. The peak at 1031 cm^−1^ are fingerprint regions related with pentasubstituted benzene rings of tannic acid [36].

### 2.4. Mechanical Properties

**Figure 4:**
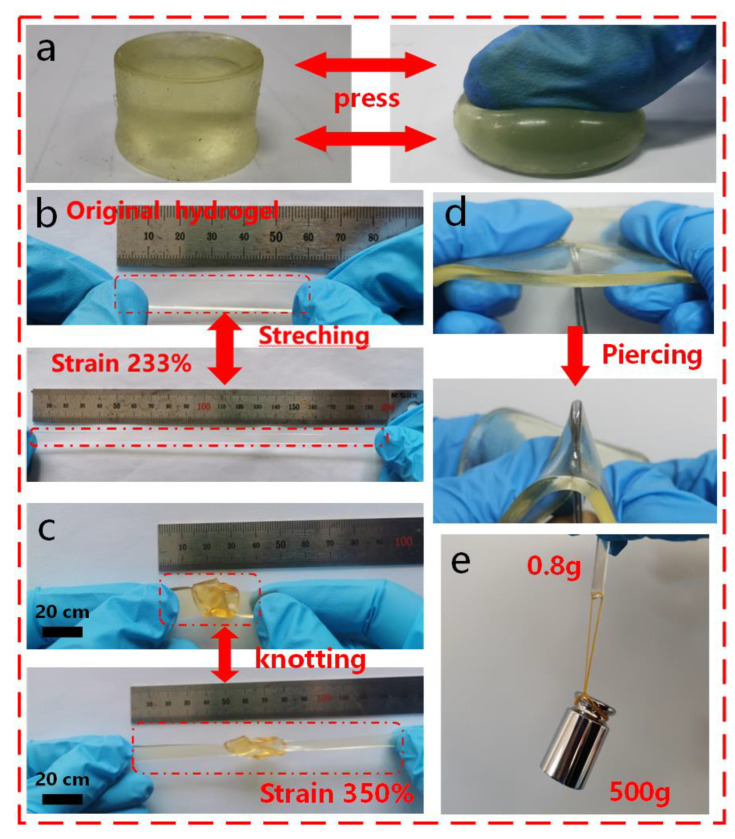
PGTA organohydrogels exhibited excellent mechanical properties: (**a**) reversible compression; (**b**) reversible stretching; (**c**) knotted stretching; (**d**) puncture resistance; (**e**) length change of the hydrogel when 500 g weight was applied.

The mechanical properties of the organohydrogels in compression, stretching, knotting stretching, and puncture resistance were studied by tensile tests. Mechanical properties are an important feature of flexible electronics, endowed with operationalization of the internal conditions and mechanical deformation of electronic devices during practical use. As shown in Figure 4, due to the combination of double network and synergistic dynamic physical crosslinking [37], the endowed PGTA organohydrogels exhibited excellent mechanical properties. The organohydrogels could be largely reversibly compressed (Figure 4a) and stretched (Figure 4b). Even when knotted and deformed, the organohydrogels could be cyclically stretched to several times their original length without breaking (Figure 4c). The outstanding flexible stretchability, fatigue resistance, and self-healing cycle stability of organohydrogels are exemplified. In addition, the organohydrogels also had excellent puncture resistance (Figure 4d) and the hydrogels could also withstand a weight of 500 g (Figure 4e), which is equivalent to about 625 times their weight. Therefore, PGTA ionic organohydrogels can serve as an important component of wearable strain hydrogel sensors due to their excellent mechanical properties.

**Figure 5:**
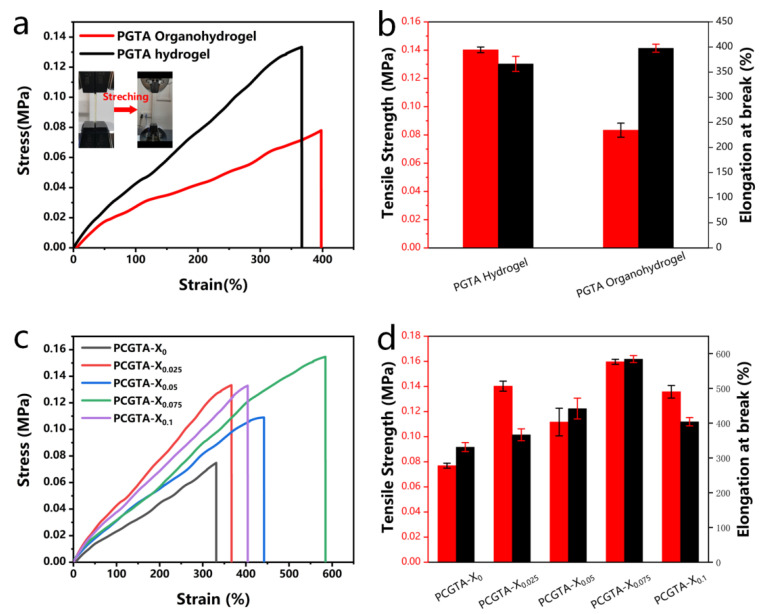
(**a**) Strain–stress curves of PGTA organohydrogels and PGTA hydrogels; (**b**) tensile strength and elongation at break; (**c**) strain–stress curves of PCGTA organohydrogels with varying CNC contents; (**d**) tensile strength and elongation at break.

The mechanical properties of the organohydrogels were studied by a universal testing machine. Figure 5 shows the effect of the content of organic solvent and CNC on the mechanical properties of the hydrogel. First, the tensile strength of PGTA hydrogel was 0.41 MPa, and the elongation at break was 366.54%. Compared with PGTA hydrogel, both the tensile strength and elongation at break produced relative changes, the tensile strength of PGTA organohydrogel was 0.08 MPa, and the elongation at break was 397.71% (Figure 5a,b). This is due to the formation of hydrogen bond entanglement between glycerol and water molecules, the increase of crosslink density, solid content, and physical crosslinking, which improve the mechanical properties of organohydrogels. On the other hand, the elongation at the break of the organohydrogels increased with the increase of CNC content. This is due to the strong ionic reaction between Al^3+^ and the amino ions of gelatin. With the increase of CNC content, the tensile strength and elongation at the break of organohydrogels increased to the peak, and the tensile strength and elongation at break increased to 0.16 MPa and 584.35%. However, when they broke through the critical point, this led to a sudden decrease in tensile strength and elongation at break (Figure 5c,d), which may be caused by too many crosslinking points in the hydrogel. According to the above results, the organohydrogels exhibited good mechanical properties, which are crucial for the potential applications of organohydrogel-based wearable sensor electronics.

### 2.5. Self-Adhesiveness

**Figure 6:**
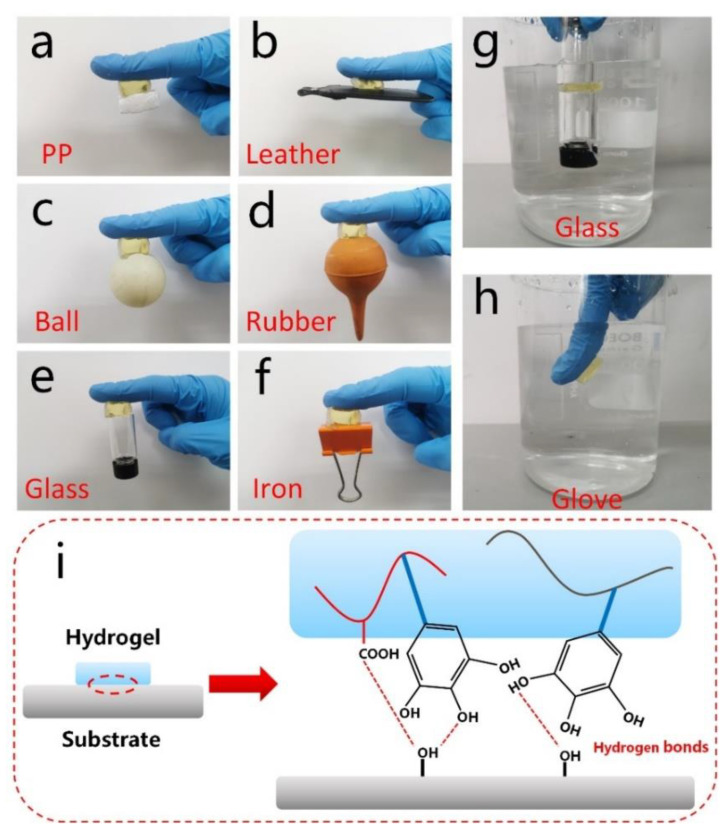
Adhesive properties of PGTA organohydrogels. Organohydrogels can be bonded on various surfaces, including (**a**) PP; (**b**) leather; (**c**) ball; (**d**) rubber; (**e**) glass; (**f**) iron; (**g**,**h**) self-adhesiveness of the glue to glass and laboratory gloves in water; (**i**) schematic illustration of the adhesion mechanism of organohydrogels.

Reliable adhesion is one of the essential functions required for hydrogel-based wearable sensors. The presence of a large number of pyrogallol groups on tannic acid (TA) imitates the polyphenolic properties of mussel photoproteins, and this process can mimic the adhesion mechanism of mussels [38]. On the other hand, pyrogallol groups are introduced by TA. The phenolic group interacts with the free carboxyl groups on the poly(acrylic acid) (PAA) chain, and through this interaction (including hydrogen bond crosslinking, metal complexation, and hydrophobic interaction), PGTA organohydrogels are produced on various substrates with strong adhesion [39,40]. As shown in Figure 6, PGTA organohydrogels exhibited unique long-lasting self-adhesive properties to various substrate surfaces, including PP, leather, a ball, rubber, glass, and iron (Figure 6a–f), among others. In addition, the organohydrogel formed an adhesive surface between the two glass surfaces, bearing the corresponding weight, respectively (Figure 6g), and without falling off in water (Figure 6h). It is worth noting that the wearable sensor will form a stable contact with the biological epidermal tissue under special circumstances to improve the accuracy and stability of electronic signal monitoring. Finally, Figure 6i is a schematic diagram of the adhesion mechanism of organohydrogels. In this network, the pyrogallol group of TA and the carboxyl group on the PAA chain act synergistically, which can rapidly form noncovalent bonds (such as hydrogen bonds and metal coordination) with other functional groups in different substrates. Altogether, the compliance of organohydrogels with normal surfaces, abnormal surfaces, and adhesion under special wet conditions improves the lifetime of hydrogel sensors.

### 2.6. Antidrying Property

**Figure 7:**
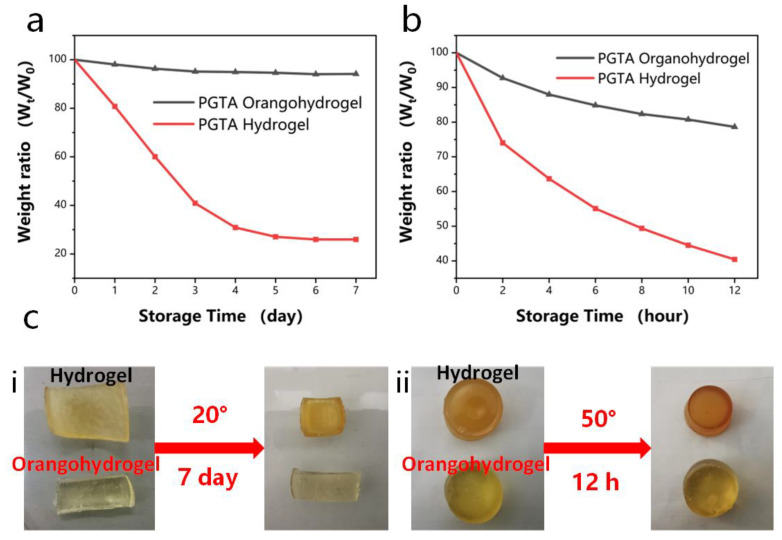
(**a**) Weight change of PGTA organohydrogel and PGTA hydrogel after 7 days of storage at 20 °C; (**b**) weight change of PGTA organohydrogel and PGTA hydrogel after 12 h of storage at 50 °C; (**c**) (**i**,**ii**) comparison of pictures of PGTA organohydrogel and PGTA hydrogel after storage under different conditions.

The unavoidable evaporation of water molecules in traditional hydrogels under ambient conditions severely weakens the performance of hydrogel sensors. For hydrogel-based sensors, achieving water retention and long-term durability is still a challenge. PGTA organohydrogel adopts a water/glycerol binary solvent system, and glycerol has properties, such as being colorless, non-toxic, and moisturizing. It has favorable properties for surface moisture storage, biocompatibility, and wound healing [41]. As shown in Figure 7a, the PGTA hydrogel and PGTA organohydrogel were stored under normal conditions at room temperature (20 °C) for seven days, and the weights were recorded. Due to the evaporation of water molecules, the weight of PGTA hydrogel began to decrease on the second day, and only 25.97% of the weight remained after seven days. However, the PGTA organohydrogel remained wet (over 90%) after seven days. This is due to the hygroscopic and moisturizing effects of glycerin in the air, which makes the dynamic balance of water molecules in glycerin difficult to be broken.

In addition, the weight of PGTA organohydrogel remained above 80% of the original, but the residual weight ratio of PGTA hydrogel was only 40% (Figure 7b). Due to the rapid evaporation of water molecules at a high temperature of 50 °C, glycerin and water molecules are entangled and crosslinked to form hydrogen bonds, which hinders the evaporation of water molecules in the state. The dimensions of the PGTA organohydrogels remained in their original shape after storage for seven days. In contrast, PGTA hydrogels became smaller in size and showed curled morphology (Figure 7c). Based on the above results, the water/glycerol binary solvent system endows organohydrogels with long-term use and durability. Compared with conventional hydrogels, this facile method ensures the durability of organohydrogels in further applications.

### 2.7. Ionic Conductivity

**Figure 8:**
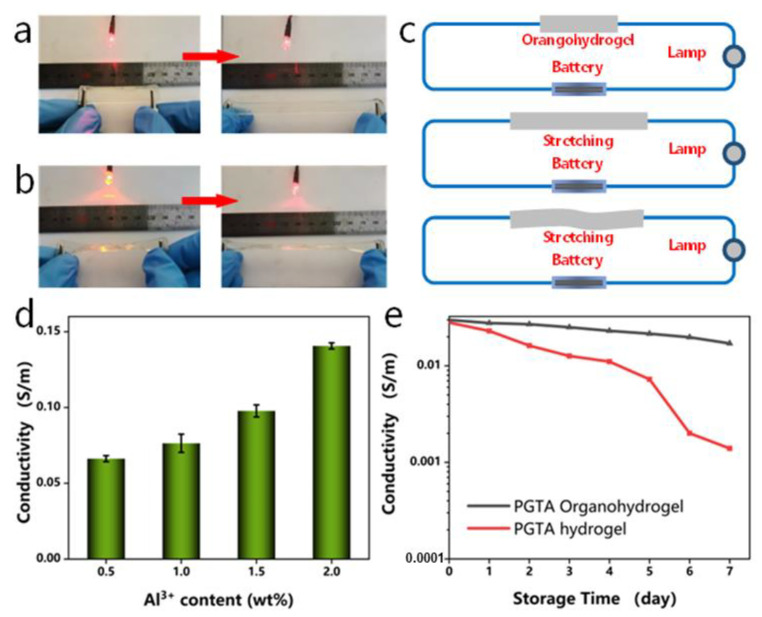
Circuit consisting of LED lights connected to conductive PGTA organohydrogels in different states, including (**a**) original length and stretch and (**b**) deformed stretch; (**c**) schematic diagram of the circuit with the organohydrogel as a conductor at a constant voltage of 3 V; (**d**) ionic conductivity of PGTA organohydrogel at different concentrations of Al^3+^ (0.5–2 wt%); (**e**) ionic conductivity of PGTA organohydrogel and PGTA hydrogel after storage at 20 °C for 7 days.

The introduction of Al^3+^ and Cl^−^ in the PGTA organohydrogel provided it with conductive properties, and the electrical properties of the hydrogels under different conditions were studied; the results are shown in Figure 8. The organohydrogels were used as conductors at a constant voltage of 3 V to enable LED diodes to emit light under mechanical deformation (Figure 8a,b). This shows the excellent conductivity of the ionic conductive hydrogels (Figure 8c).

In addition, compared with PGTA-Y_0.5_ organohydrogel, the ionic conductivity of PGTA-Y_2_ increased twofold with the stepwise increase of Al^3+^ concentration, and the conductivity of the organohydrogels increased from 0.07 S/m to 0.14 S/m (Figure 8d). This is because the carboxyl groups on the surface of organohydrogels can attract ions and provide more hopping sites for ion transfer [42]. These results demonstrate that the conductivity of organohydrogels may be related to the amount of conducting ions. Additionally, the ionic conductivity of PGTA organohydrogel and PGTA hydrogel at room temperature of 20 °C was investigated. The organohydrogels still retained excellent electrical conductivity after being stored at room temperature of 20 °C for 7 days. However, the common hydrogel shrank severely, and the ionic conductivity showed a linear decreasing trend (Figure 8e). In summary, the introduction of metal ions provides a conductive network for the hydrogel structure, which improves the electrical performance and stability of the hydrogel sensor.

### 2.8. Sensing Performance

**Figure 9:**
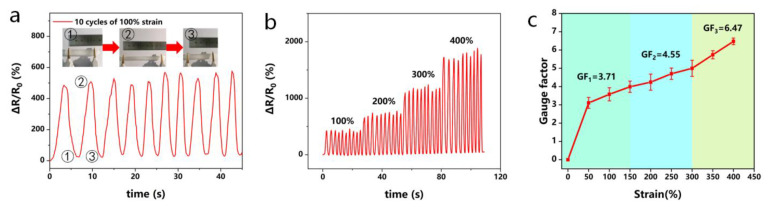
Demonstration of PGTA organohydrogels as strain sensors under tensile deformation: (**a**) relative resistance change rate of the strain sensor under 100% cyclic tensile strain; (**b**) relative resistance change rate of the strain sensor under 0–400% cyclic strain; (**c**) variation of GF with tensile strain.

The sensitivity factor (GF) can provide a relatively stable assessment of the strain sensitivity of hydrogels to external shape changes and is calculated from the slope of the relative resistance (R-R_0_/R_0_) versus strain. The fitted linear relationship between the relative resistance change rate and tensile strain is an important property of hydrogel strain sensors. It can be seen from Figure 9 that the hydrogel sensor could record stable electrical signals (Figure 9a,b) in ten cycles of stretching and 100–400% stretching, converting mechanical deformation into electrical signals. Notably, no significant loss of electrical signal or signs of conductivity weakening were observed during several successive cycles of loading and unloading during strains ranging from 100–400%. In addition, Figure 9c shows the variation curve of GF with strain, which can be divided into three linear response regions (0–150%, GF = 3.71; 150–300%, GF = 4.55; 300–400%, GF = 6.47). This linear response also shows that the sensor presents monotonicity in the relationship between strain and resistance change, and is not disturbed by external factors, which ensures the reliability of the signal.

**Figure 10:**
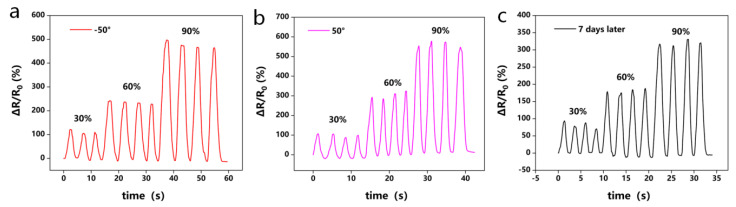
PGTA organohydrogels as environmental strain-resistant sensors at different temperatures: relative resistivity change of hydrogel at (30–90%) strain after (**a**) storage at −50 °C; (**b**) storage at 50 °C for 6 h; (**c**) storage at room temperature for 7 d.

Environmental resistance is one of the biggest obstacles limiting the practical application of hydrogel wearable sensors. Therefore, the sensing performance of the PGTA organohydrogel sensors after storage in harsh environments for 6 h (−50 °C and 50 °C) and after storage at room temperature for 7 days was tested. As shown in Figure 10, all the sensors exhibited excellent stability even in harsh environments (Figure 10a,b) and after 7 days of storage (Figure 10c), the ability to detect stable electrical signals, and reversibility; furthermore, resistance returned to its initial value after a change. Similarly, the resistive signal of the organohydrogel sensor could remain almost constant during strain cycles, which makes it suitable for use in wearable electronics under extremely harsh conditions. This can be attributed to the following reasons: the rapid dissociation of physical interactions including metal ion Al^3+^ coordination bonds and hydrogen bonds in the organohydrogel; two water molecules in the hydrogel will combine with one glycerol molecule, which can form strong hydrogen bonds; there are still some unbound water molecules in the hydrogel under high temperature conditions, and these unbound free water molecules will evaporate rapidly under high temperature conditions. In conclusion, organohydrogel sensors exhibit excellent signal sensing capabilities under different conditions.

### 2.9. Human Motion Detection

**Figure 11:**
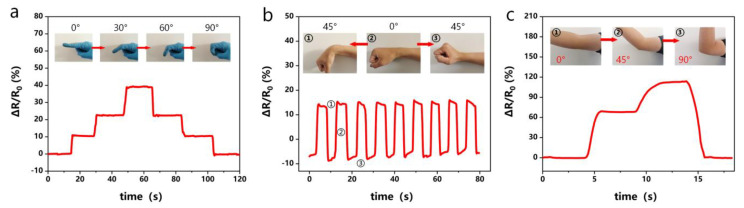
PGTA organohydrogels as strain-sensitive wearable sensors for human motion monitoring: (**a**) finger flexion; (**b**) wrist flexion; (**c**) elbow flexion.

As shown in Figure 11, hydrogels can be easily fabricated into wearable strain sensors (electronic devices) for monitoring human motion signals. Hydrogel sensors can be directly attached to human joints, including fingers, wrists, and elbows, to effectively convert human motion behavior into electrical signals in real time. According to the bending behavior of the finger at different angles (0, 30, 60, and 90°), the relative resistance process increases first and then decreases (Figure 11a), which is caused by the stretching of the strain sensor. Furthermore, motion behaviors through different joints were precisely recorded and distinguished according to patterns distinguishable by relative changes in the relative resistance curves (Figure 11b,c). The wearable sensors attached to the wrist and elbow sensed motion in real time, and the output electronic signals clearly show the intensity and frequency of motion, demonstrating the excellent strain sensitivity of organohydrogel-based wearable sensors. Based on these results, organohydrogels can be considered a promising wearable device platform for the behavioral detection of human motion.

## 3. Experimental Section

### 3.1. Materials

Gelatin (Gel) was purchased from Sinopharm Chemical Reagent Co., Ltd., Cellulose nanocrystals (CNC) (Shanghai, China) were acquired from Guilin Qihong Technology Co., Ltd., (Guilin, China). Acrylic acid (AA), glycerol (Gly), tannic acid (TA), aluminum chloride hexahydrate (AlCl_3_·6H_2_O), and ammonium persulfate (APS) were purchased from Shanghai Titan Scientific Co., Ltd., (Shanghai, China). N,N′-Methylene-bisacrylamide crosslinker (MBA) was purchased from Shanghai Macklin Biochemical Co., Ltd., (Shanghai, China). All reagents were used directly without further purification.

### 3.2. Preparation of the PCGTA Organohydrogels

PCGTA organohydrogels were synthesized by a one-pot method and ultraviolet photopolymerization. First, AA (3.2 g), Gel (0.8 g), TA (0.04 g), MBA (0.1 g), APS (0.03 g), a series of different concentrations of AlCl_3_·6H_2_O (respectively, 0.5, 1, 1.5, and 2 wt%), and CNC (respectively, 0.025, 0.05, 0.075, and 0.1 wt%) were dissolved in deionized water/glycerol (1:1). The mixed solution was vigorously stirred at 60 °C for 3 h with a constant temperature heating magnetic stirrer. The resulting clear solution was then transferred to a PTFE mold (80 mm × 10 mm × 2 mm) and cooled at room temperature for 0.5 h. Subsequently, it was transferred to a UV ultraviolet curing lamp (20 W 365 nm) for a 0.5 h photocuring reaction to form PCGTA organohydrogel. The first group of CNC with different contents is represented as PCGTA-X organohydrogels, and the second group of Al^3+^ with different concentrations is represented as PGTA-Y organohydrogels. In addition, the PGTA hydrogel without glycerol was prepared as a comparison. The composition of the organohydrogels is shown in Table 1.

### 3.3. Scanning Electron Microscope

Samples of different PCGTA hydrogels were processed by freeze-drying to obtain dehydrated hydrogels. The microscopic morphology of the hydrogel was tested with a scanning electron microscope (SEM, JSM-7600F) at 15 kV.

### 3.4. Fourier Transform Infrared Spectroscopy

The hydrogel samples were dehydrated by Freezing Drier. Fourier transform infrared spectroscopy (FTIR, CX-9600) was used to test the chemical structure of the samples, and the group changes of the samples were analyzed according to the absorption area of the sample groups. The resolution was ±2 cm^−^^1^, the wavenumber range was 4000–500 cm^−^^1^, and the step size was 4 cm^−^^1^.

### 3.5. Mechanical Properties

Mechanical tests were performed using a universal material tensile testing machine (CMT5504, MTS Systems, Shanghai, China) equipped with a 2 KN loading unit. The organohydrogels were cut into long strips (60 mm × 10 mm × 2 mm) and subjected to tensile tests at a loading rate of 20 mm/min. All samples were tested five times and the average value was taken. There was no time interval between each test in the experiment, and the elongation at break and tensile strength were obtained, respectively, through the stress–strain curve.

### 3.6. Adhesion Tests

The adhesion performance of PGTA organohydrogel was tested by lap shear test. The hydrogel sample was cut into a rectangular specimen (thickness 5 mm) and placed between the surfaces of two materials (PP, leather, a ball, rubber, glass, iron, and glove) to test the adhesive properties of the hydrogel.

### 3.7. Moisture Retention Property Tests

PGTA organohydrogel and PGTA hydrogel were cut to the same size. The hydrogel samples were kept at room temperature for 7 days (recorded every 1 day) and at 50 °C for 12 h (recorded every 2 h). The formula for calculating the weight ratio is:

(1) $$Weight\;ratio=\frac{W_{t}}{W_{0}}\times 100\%$$

where W_t_ is the weight of the hydrogel after t h/day and W_0_ is the initial weight of the hydrogel.

### 3.8. Ionic Conductivity Measurement

PGTA organohydrogels were cut into long strips (40 mm × 10 mm × 2 mm). LCR digital bridge (LCR TH2832, Changzhou, China) was used to measure the resistance change of hydrogels with different concentrations of Al^3+^ in real time, and to calculate the ionic conductivity (σ) of the hydrogels. In addition, the hydrogels were subjected to a cyclic tensile test (100–400%) by using a universal material testing machine to record the relative resistance change of the hydrogels under tensile strain.

The ionic conductivity (σ) of the organohydrogels was calculated by the following formula:

(2) $$\sigma =\frac{L}{R\times S}$$

where σ represents the ionic conductivity of the hydrogel (S/m), L represents the length of two adjacent electrodes of the hydrogel (m), R represents the resistance value of the hydrogel (Ω), and S is the contact area of the hydrogel (m^2^).

### 3.9. Electrical Measurements

The strain sensitivity of the organohydrogels was tested and evaluated by the gauge factor (GF), which tested the stretching cycle, electrical stability, and environmental resistance (−50°, 50°, and after 7 days) of the organohydrogel sensors. LCR digital bridge tester (LCR TH2832) was used to test the relative resistance change (ΔR/R_0_) and GF of the stretching/release cycle of the hydrogel sensors. The relative resistance change of the hydrogels was calculated by the following formula:

(3) $$Relative\;resistance\;change=\frac{R-R_{0}}{R_{0}}\times 100\%$$

where R_0_ and R represent the electrical resistance (Ω) before and after tensile strain, respectively.

The GF of the hydrogel was calculated by the following formula:

(4) $$GF=\frac{\frac{R-R_{0}}{R_{0}}}{\frac{l-l_{o}}{l_{0}}}$$

where l_0_ and l represent the length (m) of the organohydrogels after strain and before strain, respectively.

### 3.10. Human Motion Detection Demonstration

This experiment was completed with the assistance of one volunteer; images and data were released after obtaining informed consent. The hydrogel sensors were encapsulated with polyimide tape and assembled into flexible wearable strain sensors that adhered to different joints of the human body, such as fingers, wrists, and elbows, and measured the resistance changes caused by different parts in real time.

## 4. Conclusions

In summary, a stretch-adhesive ionic organohydrogel was prepared by a one-pot water/glycerol binary solvent dispersion method and UV-illumination. It has the characteristics of high water retention, frost resistance, long-term stability, high stretchability, strong adhesion, and repeatable cycle. The surface of the organohydrogel has a three-dimensional porous network structure. With the increase of cellulose nanocrystals content, the tensile strength (0.16 MPa) and elongation at break (584.35%) of the hydrogel increased, but it would break through the critical point and lead to a decrease. The organohydrogel exhibited reversible adhesive properties to various substrates. Due to the presence of hydrogen bonds between the binary solvent system and water molecules, which inhibit the formation of ice crystal lattices at low temperatures and hinder the divergence of water storage under various conditions, organohydrogels exhibit good antifreeze and long-lasting moisturizing properties. Furthermore, the organohydrogel sensor exhibited sensing performances at temperature range (−50 °C–50 °C) even after long-term storage (7 days). Thus, it may be an ideal wearable strain or pressure sensor device to distinguish various human motions, such as the bending behavior of different joints, due to its high sensitivity (Gauge factor = 6.47, strain: 400%). In conclusion, this poly(acrylic acid)/gelatin based hydrogel sensor has excellent stretchability, adhesion, freeze resistance, and desiccation resistance, and we believe that the developed hydrogel has good potential for high-performance wearable devices in low-temperature environments.

## Figures and Tables

**Table 1:** Composition of the organohydrogels.

Samples	Gel	AA	TA	AlCl_3_·6H_2_O	H_2_O	Gly
PCGTA-X_0_ organohydrogel	0.8 g	3.2 g	0.04 g	0.3 g	7.83 g	7.83 g
PCGTA-X_0.025_ organohydrogel	0.8 g	3.2 g	0.04 g	0.3 g	7.83 g (With 5 g CNC 0.025 wt%)	7.83 g
PCGTA- X_0.05_ organohydrogel	0.8 g	3.2 g	0.04 g	0.3 g	7.83 g (With 5 g CNC 0.05 wt%)	7.83 g
PCGTA-X_0.075_ organohydrogel	0.8 g	3.2 g	0.04 g	0.3 g	7.83 g (With 5 g CNC 0.075 wt%)	7.83 g
PCGTA-X_0.1_ organohydrogel	0.8 g	3.2 g	0.04 g	0.3 g	7.83 g (With 5 g CNC 0.1 wt%)	7.83 g
PGTA hydrogel	0.8 g	3.2 g	0.04 g	0.3 g	15.66 g	0 g
PGTA-Y_0.5_ organohydrogel	0.8 g	3.2 g	0.04 g	0.1 g	7.83 g	7.83 g
PGTA-Y_1_ organohydrogel	0.8 g	3.2 g	0.04 g	0.2 g	7.83 g	7.83 g
PGTA-Y_1.5_ organohydrogel	0.8 g	3.2 g	0.04 g	0.3 g	7.83 g	7.83 g
PGTA-Y_2_ organohydrogel	0.8 g	3.2 g	0.04 g	0.4 g	7.83 g	7.83 g

## Data Availability

The data presented in this study are available in the article. The datasets generated during and/or analyzed during the current study are available from the corresponding author upon reasonable request.

## Sample Availability

Not available.
